# Circulating neutrophil transcriptome may reveal intracranial aneurysm signature

**DOI:** 10.1371/journal.pone.0191407

**Published:** 2018-01-17

**Authors:** Vincent M. Tutino, Kerry E. Poppenberg, Kaiyu Jiang, James N. Jarvis, Yijun Sun, Ashish Sonig, Adnan H. Siddiqui, Kenneth V. Snyder, Elad I. Levy, John Kolega, Hui Meng

**Affiliations:** 1 Toshiba Stroke and Vascular Research Center; University at Buffalo, State University of New York, Buffalo, New York, United States of America; 2 Department of Biomedical Engineering, University at Buffalo, State University of New York, Buffalo, New York, United States of America; 3 Genetics, Genomics, and Bioinformatics Program, University at Buffalo, State University of New York, Buffalo, New York, United States of America; 4 Department of Pediatrics, University at Buffalo, State University of New York, Buffalo, New York, United States of America; 5 Department of Microbiology and Immunology, University at Buffalo, State University of New York, Buffalo, New York, United States of America; 6 Department of Computer Science and Engineering, University at Buffalo, State University of New York, Buffalo, New York, United States of America; 7 Department of Neurosurgery, University at Buffalo, State University of New York, Buffalo, New York, United States of America; 8 Department of Radiology, University at Buffalo, State University of New York, Buffalo, New York, United States of America; 9 Department of Neurology, University at Buffalo, State University of New York, Buffalo, New York, United States of America; 10 Department of Pathology and Anatomical Sciences, University at Buffalo, State University of New York, Buffalo, New York, United States of America; 11 Department of Mechanical & Aerospace Engineering, University at Buffalo, State University of New York, Buffalo, New York, United States of America; Stellenbosch University Faculty of Medicine and Health Sciences, SOUTH AFRICA

## Abstract

**Background:**

Unruptured intracranial aneurysms (IAs) are typically asymptomatic and undetected except for incidental discovery on imaging. Blood-based diagnostic biomarkers could lead to improvements in IA management. This exploratory study examined circulating neutrophils to determine whether they carry RNA expression signatures of IAs.

**Methods:**

Blood samples were collected from patients receiving cerebral angiography. Eleven samples were collected from patients with IAs and 11 from patients without IAs as controls. Samples from the two groups were paired based on demographics and comorbidities. RNA was extracted from isolated neutrophils and subjected to next-generation RNA sequencing to obtain differential expressions for identification of an IA-associated signature. Bioinformatics analyses, including gene set enrichment analysis and Ingenuity Pathway Analysis, were used to investigate the biological function of all differentially expressed transcripts.

**Results:**

Transcriptome profiling identified 258 differentially expressed transcripts in patients with and without IAs. Expression differences were consistent with peripheral neutrophil activation. An IA-associated RNA expression signature was identified in 82 transcripts (p<0.05, fold-change ≥2). This signature was able to separate patients with and without IAs on hierarchical clustering. Furthermore, in an independent, unpaired, replication cohort of patients with IAs (n = 5) and controls (n = 5), the 82 transcripts separated 9 of 10 patients into their respective groups.

**Conclusion:**

Preliminary findings show that RNA expression from circulating neutrophils carries an IA-associated signature. These findings highlight a potential to use predictive biomarkers from peripheral blood samples to identify patients with IAs.

## Introduction

Intracranial aneurysm (IA) rupture can be fatal. An estimated 5% of Americans harbor unruptured IAs [1]. Because most unruptured aneurysms are asymptomatic, they remain dormant, often being found after rupture. Early IA detection would enable monitoring and treatment to prevent rupture and its devastating sequelae. Currently, most unruptured IAs are detected incidentally by cerebral imaging performed for other reasons. However, imaging is unsuitable for general IA screening due to potential risks and high costs [2]. Even for high-risk individuals (e.g., those with a family history of IA), it is debated whether imaging is cost-effective [2, 3]. Noninvasive and inexpensive strategies such as blood testing could offer a diagnostic alternative.

Recent studies have correlated RNA expression differences in the circulating blood with vascular diseases, such as thoracic aortic aneurysm [4]. We hypothesized that circulating neutrophils carry transcriptional signatures of IAs. Our rationale was that aneurysmal lesions are associated with persistent vascular wall inflammation, which can involve neutrophil responses [5]. Results from genome wide association studies suggest that IA-associated genes are involved in endothelial function, extracellular matrix maintenance, and inflammatory responses [6, 7]. Furthermore, gene expression profiling of IA tissue has demonstrated increased presence of inflammatory cytokines and chemoattractant proteins in the aneurysm wall [8, 9]. The presence of the neutrophil proteins, neutrophil gelatinase-associated lipocalin (NGAL), and myeloperoxidase (MPO) is consistent with a direct role of neutrophils in the degeneration of the vessel wall during IA natural history [10, 11]. As circulating neutrophils have been shown to display unique expression differences in other diseases characterized by inflammation [12], a continual interaction between neutrophils and an IA could leave imprints on the RNA expression of circulating neutrophils.

In this study, we investigated whether neutrophils have different RNA expression profiles in patients with IAs compared to patients without IAs. We recruited patients with and without aneurysms (confirmed on angiography) and paired them based on demographics and comorbidities. Next-generation RNA sequencing of circulating neutrophils was performed to identify an IA-associated expression signature in their transcriptomes. We further assessed if the IA-associated expression signature could distinguish patients with and without IA in a heterogeneous independent cohort of patients. Gene ontology analysis and physiological pathway modeling were used to determine the biological function of differentially expressed transcripts in IA. Results from this study could motivate future efforts towards developing blood-based biomarkers and shed light on the pathophysiology of IAs.

## Materials and methods

### Clinical study

This study was approved by our institutional review board (study no. 030–474433). Methods were carried out in accordance with the approved protocol. Written informed consent was obtained from all subjects. Between November 2013 and May 2014, 77 peripheral blood samples were collected from patients undergoing cerebral digital subtraction angiography (DSA) at our institute: 35 patients had a positive IA diagnosis and 42 had a negative IA diagnosis (controls). Positive or negative IA diagnosis was confirmed by imaging, and patient medical records were collected for pairing patients with IAs to controls. Additionally, each patient’s complete blood count, which was taken within 3 months of blood collection, was recorded.

Patients undergoing cerebral digital subtraction angiography (DSA) with positive and negative intracranial aneurysm (IA) diagnoses were enrolled in this study. Reasons for the patients to receive DSA included confirmation of findings from noninvasive imaging of the presence of IAs, vascular malformations, or carotid stenosis or follow-up noninvasive imaging of previously detected IAs. All consenting patients were older than 18 years, were English speaking, and had not received previous treatment for IA. To ensure that differences in the circulating neutrophils were not influenced by inherent inflammatory conditions, we excluded patients who potentially had altered leukocyte transcriptomes; this included patients who were pregnant, had recently undergone invasive surgery, were undergoing chemotherapy, had a body temperature above 37.78°C (100oF), had received solid organ transplants, had autoimmune diseases, and those who were taking prednisone or any other immunomodulating drugs. Furthermore, the included patients did not have any other known cerebrovascular malformations or extracranial aneurysms, including abdominal aortic aneurysms.

### Sample preparation

Sixteen mL of blood was drawn from the access catheter in the femoral artery and transferred into two 8 mL, citrated, cell preparation tubes (BD, Franklin Lakes, NJ). Neutrophils were isolated within 1 hour of peripheral blood collection, as described elsewhere [12]. Cell preparation tubes were centrifuged at 1,700 × *g* for 25 minutes to separate erythrocytes and neutrophils from mononuclear cells and plasma in the peripheral blood samples. Erythrocytes and neutrophils were collected into a 3 mL syringe and placed into an erythrocyte lysis buffer that was made in-house. After all erythrocytes were lysed, the neutrophils were isolated by centrifugation at 400 × *g* for 10 min and disrupted and stored in TRIzol reagent (Life Technologies, Carlsbad, CA) at -80 oC until further processing. Neutrophils isolated in this fashion are more than 98% CD66b+by flow cytometry and contain no contaminating CD14+ monocytes [13].

Total neutrophil RNA was extracted using TRIzol, according to the manufacturer’s instructions. Trace DNA was removed by DNase I (Life Technologies, Carlsbad, CA) treatment. The RNA was purified using the RNeasy MinElute Cleanup Kit (Qiagen, Venlo, Limburg, Netherlands) and suspended in RNase-free water. After RNA isolation, the purity and concentration of RNA in each sample was measured by absorbance at 260 nm on a NanoDrop 2000 (Thermo Scientific, Waltham, MA), and 200–400 ng of RNA was sent to our university’s Next-Generation Sequencing and Expression Analysis Core facility for further quality control. Precise RNA concentration was measured at the core facility via the Quant-iT RiboGreen Assay (Invitrogen, Carlsbad, CA) with a TBS-380 Fluorometer (Promega, Madison, WI), whereas the quality of the RNA samples was measured with an Agilent 2100 BioAnalyzer RNA 6000 Pico Chip (Agilent, Las Vegas, NV). RNA samples with 260/280 ≥ 1.9 and an RNA integrity number (RIN) ≥ 6.0 were considered for RNA sequencing.

### Cohort creation

Before sequencing, samples from IA patients and control subjects were paired by demographics and comorbidities to control for confounding variables. First, samples that did not have acceptable RNA quality for sequencing were excluded. Next, each patient in the IA group was paired with a control subject by factors that have been reported in the literature to correlate with IA. These included (in order of decreasing importance) age, sex, smoking status (yes or no), presence of hypertension, presence of hyperlipidemia, and presence of heart disease [14]. Matching criteria also included stroke history, presence of diabetes mellitus, and presence of osteoarthritis, when possible. With the exception of age, the factors used for matching were quantified as binary data points. The clinical factors were retrieved from the patients’ medical records via the latest Patient Medical History form (see S1 Fig) administered prior to imaging. Since this form in the medical record contained self-reported information, the presence of each comorbidity was corroborated with each patient’s reported list of medications (e.g., hypertension with lisinopril, hyperlipidemia with simvastatin, heart disease with metoprolol, stroke history with clopidogrel, diabetes mellitus with metformin, and osteoarthritis with nonsteroidal anti-inflammatory drugs/tramadol). We were able to corroborate 84% of the clinical data points for patients’ comorbidities through their medication history.

After performing the original experiments to identify an IA-associated neutrophil expression signature, we used the same clinical protocol to recruit an additional 5 patients with IAs and 5 IA-free controls into a small replication cohort (n = 10) to test whether the IA-associated signature could separate IA patients from controls in the second cohort. Blood samples and RNA were handled in the same manner as those in the original cohort, and the same RNA sequencing and data analysis protocols were followed. However, prior to sequencing, we did not control for demographics and comorbidities to obtain a more heterogeneous cohort.

### RNA sequencing

RNA libraries for these samples were constructed at our university’s Next-Generation Sequencing and Expression Analysis Core facility using the TruSeq RNA Library Preparation Kit (Illumina, San Diego, CA). All samples were subjected to 50-cycle, single-read sequencing in the HiSeq2500 (Illumina) and were demultiplexed using Bcl2Fastq v2.17.1.14 (Illumina). Gene expression analysis was completed using the Tuxedo Suite. Short RNA fragment data were compiled in FASTQ format and aligned to the human reference genome (human genome 19 [hg19]) using TopHat v2.1.13 [15]. Gene expression levels were calculated using fragments per kilobase of transcript per million mapped reads (FPKM) normalization in CuffLinks v2.2.1 [15]. RNA sequencing data files and processed transcript expression have been made available at NCBI’s GEO (accession no. GSE106520). To evaluate the quality of RNA sequencing, we performed quality control analysis using both FASTQC [16] before alignment and MultiQC [17] after alignment.

### Differential expression analysis

Differential gene expression analysis was performed in CuffDiff v2.2.1 [18] and visualized in the CummeRbund v2.7.1 package in R [15, 19]. We used CuffDiff v2.2.1 (Trapnell Laboratory), which compared the log ratio of FPKM values in the IA and control groups against the log ratio of FPKM values of the IA group, and computed a test statistic [20]. The test statistic was calculated using the negative binomial distribution to model the variance of each sample and the square root of the Jensen-Shannon divergence to assess differences in relative abundance. The change in Jensen-Shannon divergence was then assigned a p-value, as described elsewhere [20].

Transcripts were considered significantly differentially expressed at p<0.05. We defined an IA-associated expression signature as those significant transcripts that also had an absolute fold-change ≥2. *Post hoc* power estimation was performed following Hart et al. [21] with α = 0.05, an average coefficient of variation of 0.404 (calculated from FPKMs), and counts per million mapped reads of 38. Multiple testing correction was performed by using the Benjamini-Hochberg method [22], and q-values were reported for each transcript.

### Verification by RT-qPCR

To verify expression differences measured by RNA sequencing, quantitative reverse transcription polymerase chain reaction (RT-qPCR) was performed. We verified expression difference of 5 differentially expressed transcripts (*CD177*, *SERPING1*, *GBP5*, *IL8*, *NAAA*) in order to conserve RNA material. These 5 transcripts were chosen because they were among the most prominently differentially expressed transcripts, i.e., they were highly abundant (FPKM>10) and significantly differentially expressed (p<0.05) with an absolute fold-change >1.5. For each transcript, oligonucleotide primers were designed with a ~60°C melting temperature and a length of 15–25 nucleotides to produce PCR products with lengths of 50–200 base pairs using Primer3 software [23] and Primer BLAST (NCBI, Bethesda, MD). The replication efficiency of each primer set was tested by performing qPCR on serial dilutions of cDNA samples (primer sequences, annealing temperatures, efficiencies, and product lengths are shown in S1 Table).

For reverse transcription, first-strand cDNA was generated from total RNA using OmniScript Reverse Transcriptase kit (Qiagen, Venlo, Limburg, Netherlands) according to the manufacturer’s instructions. Quantitative PCR was run with 10 ng of cDNA in 25 μ reactions in triplicate in the Bio-Rad CFX Connect system (Bio-Rad, Hercules, CA) using ABI SYBR Green Master Mix (Applied Biosystems, Foster City, CA) and gene-specific primers at a concentration of 0.02 μM each. The temperature profile consisted of an initial step of 95°C for 10 minutes, followed by 40 cycles of 95°C for 15 seconds and 60°C for 1 minute, and then a final melting curve analysis from 60°C to 95°C for 20 minutes. Gene-specific amplification was demonstrated by a single peak using the Bio-Rad dissociation melt curve. As previously described [12], *GAPDH* expression was used for normalization, and fold-changes between groups were calculated using the 2-^ΔΔ^Ct method.

### Dimensionality reduction

We performed dimensionality reduction by unsupervised principal component analysis (PCA) and multidimensional scaling (MDS) using the transcriptomes of each sample in the CummeRbund and prcomp packages in R Bioconductor under the default settings [19]. For hierarchical clustering, we used the hclust package in R [24]. Dendrograms were created using Ward linkage from z-score normalized transcript levels.

### Bioinformatics

We performed gene set enrichment analysis using the open-source software GO::TermFinder (Stanford University School of Medicine, Stanford, CA) [25]. This tool determined whether any gene ontology terms annotated two lists of genes (i.e., genes with higher expression in samples from patients with IAs than those without IA and genes with lower expression in samples from patients with IAs than those without IA) greater than what would be expected by chance. Significantly enriched ontologies were reported if the Q-Value was <0.05, based on significance testing using the hypergeometric distribution.

Networks of potential interactions were generated using Ingenuity Pathway Analysis (IPA) software (Qiagen Inc., https://www.qiagenbioinformatics.com/products/ingenuity-pathway-analysis) [26]. For IPA, each gene identifier was mapped to its corresponding gene object in the Ingenuity Knowledge Base and overlaid onto a molecular network derived from information accumulated in the Knowledge Base. Gene networks were algorithmically generated based on their “connectivity” derived from known interactions between the products of these genes. Networks were considered significant if their p-scores were >21.

## Results

### Study participants

During the 6-month study period, we collected 77 blood samples (35 from patients with IA, 42 from control subjects) as well as angiographic images and medical records data from individuals undergoing cerebral DSA. Of the blood samples collected, 37 (16 from IA patients, 21 from controls) had a sufficient quality of neutrophil RNA for sequencing. Pairing on the basis of demographics and comorbidities resulted in a final cohort of 22 individuals, including 11 IA patients and 11 IA-free controls (Table 1). These samples were of sufficient quality and had an average 260/280 of 2.02 and an average RIN of 7.04 (S2 Table). Patients with IAs had aneurysms ranging in size from 1.5–19 mm, and included 3 individuals with multiple IAs (S3 Table). There was no statistical difference in age (p>0.05, Student’s *t*-test), and other factors (p>0.05, χ^2^ test) (Table 1) as well as white blood cell populations between the two groups (p>0.05, Student’s *t*-test) (S2 Fig).

**Table 1:** Clinical characteristics*.

	Patients with IA (n = 11)	Patients without IA (n = 11)	P-Value
**Age (years) (Mean±SE)**	66.91±2.84	64.73±4.22	0.67
**Age (years) [Median (Q1/Q3)]**	67 (60.5/72)	70 (60/71.5)	
**Sex**			
*Female*	63.64%	54.55%	0.66
**Current Smoker**			
*Yes*	18.18%	18.18%	1.00
**Comorbidities**			
*Hypertension*	63.64%	81.82%	0.34
*Hyperlipidemia*	45.45%	54.55%	0.67
*Heart disease*	18.18%	27.27%	0.61
*Stroke history*	0.00%	9.09%	0.31
*Diabetes mellitus*	18.18%	36.36%	0.34
*Osteoarthritis*	27.27%	27.27%	1.00

*We controlled for demographics and comorbidities so no factor was significantly higher in patients with IA or without IA (confirmed on imaging). There is no significant difference in age (p>0.05 by Student’s t-test) sex, smoking history, and comorbidities (χ^2^>0.05, chi-squared test) between the two groups. (IA = intracranial aneurysm, SE = standard error, Q = quartile)

### Neutrophils have an IA-associated RNA expression signature

**Fig 1:**
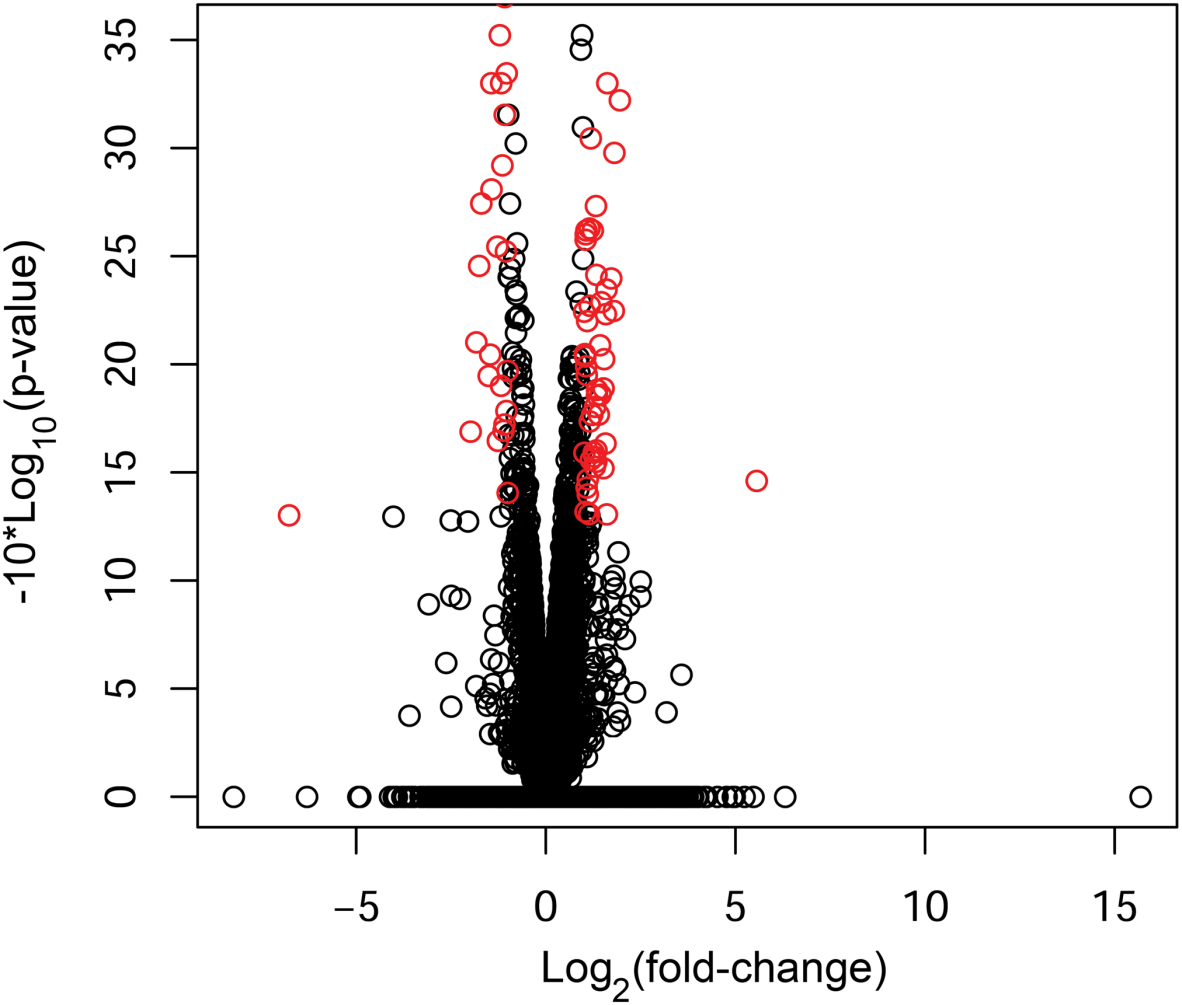
Expression differences between patients with IAs and controls, and an IA-associated expression signature. The volcano plot demonstrates differential RNA expression between the two groups. Red circles indicate an IA-associated signature of significantly differentially expressed transcripts (p<0.05) with an absolute fold-change≥2.

We performed RNA sequencing to identify differentially expressed neutrophil transcripts between 11 patients with IA and 11 paired controls. The sequencing had an average of 52.05 million sequences per sample and a 96.3% read mapping rate (% aligned) (S4 Table). The volcano plot in Fig 1 shows neutrophil expression differences between IA patients and controls in terms of average fold-change in expression and significance level. From 13,377 transcripts with testable expression differences, we identified 258 transcripts that were significantly differentially expressed (p<0.05) between the two groups. We defined an IA-associated RNA expression signature as significant transcripts that were increased or decreased by a factor of 2 or more. From the 258 transcripts, 82 met these criteria and are shown by the red circles in Fig 1 and detailed in Table 2. *Post hoc* power analysis [21] estimated that a power of 0.8 was achieved in detecting expression differences of at least 1.68 fold at α = 0.05. Therefore, our statistical criteria of p<0.05 and absolute fold-change≥2 had power >0.8 in detecting this signature.

**Table 2:** The 82-transcript intracranial aneurysm-associated gene expression signature*.

Transcript	Gene ID	Accession No.	Log_2_(F-C)	P-Value	Q-Value
*MAOA*	4128	M69226.1	5.56	0.03455	0.9999
*C21orf15*	54055	AY040090.1	2.38	0.00005	0.0836063*
*CYP1B1*	1545	NM_000104.3	2.02	0.00005	0.0836063*
*ARMC12*	221481	NM_145028.4	1.95	0.0006	0.4459
*CD177*	57126	NM_020406.3	1.81	0.001	0.585244
*OLAH*	55301	NM_018324.2	1.79	0.0057	0.9999
*CYP1B1-AS1*	285154	NR_027252.1	1.73	0.004	0.9999
*FLT3*	2322	NM_004119.2	1.63	0.00005	0.0836063*
*CD163*	9332	DQ058615.1	1.62	0.0005	0.393441
*KCNMA1*	3778	NM_001014797.2	1.61	0.050	0.9999
*DACT1*	51339	NM_016651.5	1.60	0.0045	0.9999
*FAM90A1*	55138	NM_018088.3	1.58	0.0059	0.9999
*SCT*	6343	AF244355.1	1.58	0.0232	0.9999
*LOC100131289*	100131289	NR_038929.1	1.54	0.0095	0.9999
*NOG*	9241	NM_005450.4	1.52	0.013	0.9999
*SCAMP5*	192683	NM_001178111.1	1.52	0.030	0.9999
*PTGDS*	5730	NM_000954.5	1.47	0.0052	0.9999
*KIR2DS4*	3809	NM_012314.5	1.45	0.014	0.9999
*CYP4F35P*	284233	NR_026756.	1.43	0.0082	0.9999
*XKR3*	150165	NM_001318251.1	1.41	0.00005	0.0836063*
*RPL39L*	116832	NM_052969.2	1.40	0.017	0.9999
*CDHR2*	54825	NM_001171976.1	1.35	0.013	0.9999
*ENHO*	375704	NM_198573.2	1.35	0.014	0.9999
*SLC12A7*	10723	NM_006598.2	1.35	0.00005	0.0836063*
*FLJ27354*	400761	NR_033981.1	1.34	0.025	0.9999
*DGKH*	160851	NM_152910.5	1.33	0.0039	0.9999
*SDC3*	9672	AF248634.1	1.33	0.028	0.9999
*THBS1*	7057	NM_003246.3	1.32	0.0019	0.85336
*RCVRN*	5957	NM_002903.2	1.30	0.016	0.9999
*AKR1C1*	1645	NM_001353.5	1.30	0.030	0.9999
*SCRG1*	11341	NM_001329597.1	1.28	0.027	0.9999
*NRG1*	3084	NM_013959.3	1.26	0.030	0.9999
*AK5*	26289	NM_174858.2	1.24	0.0024	0.9999
*ITGA7*	3679	NM_001144996.1	1.23	0.017	0.9999
*PAM*	5066	NM_000919.3	1.20	0.00005	0.0836063*
*LYPD2*	137797	NM_205545.2	1.19	0.028	0.9999
*PRUNE2*	158471	NM_015225.2	1.19	0.0009	0.547241
*SLC22A17*	51310	NM_020372.3	1.16	0.018	0.9999
*ADTRP*	84830	NM_001143948.1	1.16	0.0054	0.9999
*ADAMTS1*	9510	NM_006988.4	1.15	0.0024	0.9999
*ECRP*	643332	NR_033909.1	1.15	0.049	0.9999
*LOC100507387*	100507387	NR_038402.1	1.11	0.040	0.9999
*KLRC2*	3822	NM_002260.3	1.11	0.034	0.9999
*AKR1C3*	8644	NM_003739.5	1.09	0.0063	0.9999
*SEPT10*	151011	NM_144710.4	1.08	0.011	0.9999
*CYYR1*	116159	NM_001320768.1	1.08	0.037	0.9999
*TCL1A*	8115	NM_021966.2	1.07	0.0024	0.9999
*VWF*	7450	NM_000552.4	1.06	0.010	0.9999
*GNLY*	10578	NM_001302758.1	1.06	0.0025	0.9999
*C4BPA*	722	NM_000715.3	1.05	0.0027	0.9999
*LINC00482*	284185	NR_038080.1	1.05	0.048	0.9999
*KIAA1598*	57698	BC022348.1	1.04	0.0092	0.9999
*PID1*	55022	NM_017933.4	1.03	0.0090	0.9999
*SERPINF2*	5345	NM_000934.3	1.02	0.027	0.9999
*VWA8*	23078	NM_015058.1	1.01	0.0057	0.9999
*CYP4F2*	8529	NM_001082.4	-1.00	0.039	0.9999
*FADS2*	9415	NM_004265.3	-1.01	0.011	0.9999
*VLDLR*	7436	NM_003383.4	-1.03	0.0005	0.393441
*CARD17*	440068	NM_001007232.1	-1.04	0.016	0.9999
*IL8*	576	AF043337.1	-1.04	0.0001	0.148633*
*G0S2*	50486	NM_015714.3	-1.05	0.003	0.9999
*FBXW8*	26259	NM_153348.2	-1.08	0.0007	0.468195
*MFSD9*	84804	NM_032718.4	-1.08	0.0002	0.26754
*CCL23*	6368	NM_005064.5	-1.09	0.019	0.9999
*C1orf226*	400793	NM_001135240.1	-1.11	0.020	0.9999
*GBP5*	115362	NM_052942.3	-1.14	0.001	0.642096
*BATF2*	116071	NM_138456.3	-1.17	0.0005	0.393441
*FCRL5*	83416	NM_031281.2	-1.18	0.013	0.9999
*SERPING1*	710	NM_000062.2	-1.21	0.0003	0.334425
*B4GALNT3*	283358	NM_173593.3	-1.26	0.023	0.9999
*PDCD1LG2*	80380	NM_025239.3	-1.28	0.0029	0.9999
*FBN1*	2200	NM_000138.4	-1.33	0.00005	0.0836063*
*PRSS21*	10942	NM_006799.3	-1.43	0.0016	0.797475
*ETV7*	51513	NM_016135.3	-1.43	0.0005	0.393441
*SEPT4*	5414	NM_004574.4	-1.46	0.009	0.9999
*EGR2*	1959	J04076.1	-1.50	0.011	0.9999
*GBP1P1*	400759	NR_003133.2	-1.70	0.0018	0.85336
*PSORS1C3*	100130889	AB932952.1	-1.75	0.0035	0.9999
*HRK*	8739	NM_003806.3	-1.83	0.0079	0.9999
*NEB*	4703	NM_001164507.1	-1.98	0.020	0.9999
*GPC4*	2239	NM_001448.2	-2.32	0.00005	0.0836063*
*LOC730441*	207147	BC039387.1	-6.77	0.0498	0.9999

*Significantly differentially expressed transcripts with q-value<0.20 (20% FDR) are marked by “*”.

Multiple hypothesis correction identified 9 transcripts with FDR<0.20; *C21orf15*, *CYP1B1*, *FLT3*, *XKR3*, *SLC12A7*, *PAM*, *IL8*, *FBN1*, and *GPC4*. Although this correction effectively reduced the number of significant transcripts, it may be more beneficial to retain all 82 significant transcripts in the aneurysm-associated signature at this early stage of discovery. This prudence is warranted because the role of circulating neutrophils in IAs is unknown and may be highly complex. Genes tend to work together in intricate networks to carry out physiological and pathophysiological functions and influence complex traits. Therefore, individual genes in these systems that by themselves might not be significant (i.e., meet strict cutoffs of statistical tests that are not designed to find biologically relevant transcripts) could still play important roles in IA pathophysiology. To avoid missing potentially informative transcripts, we included all 82 transcripts in the IA-associated signature and in the clustering analysis.

**Fig 2:**
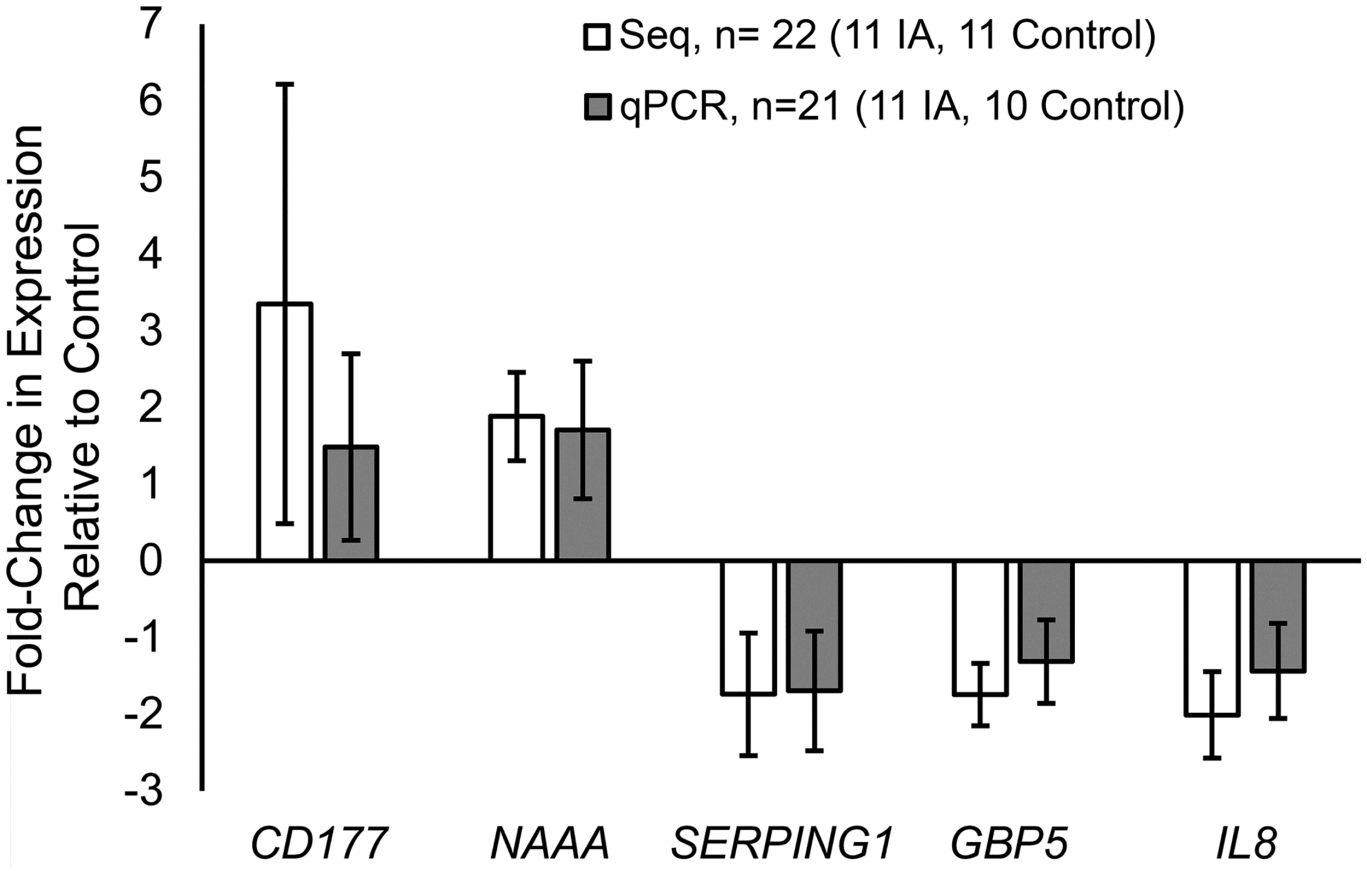
Verification of RNA expression differences by RT-qPCR. Quantitative PCR performed on 5 prominent differentially expressed transcripts demonstrates that both the magnitude and direction of the fold-change in expression measured by RNA sequencing are similar to that measured by qPCR. Only 21 samples were analyzed by RT-qPCR because sample C8 did not have enough RNA for the additional reactions. (Negative fold-change values calculated by negative inverse of fold-change, data points = average values, error bars = standard error.)

We confirmed differential expression of 5 prominent differentially expressed transcripts (*CD177*, *NAAA*, *SERPING1*, *GBP5*, and *IL8*) using RT-qPCR. Fig 2 demonstrates that the expression differences between patients with and without IA are of the same direction and of similar magnitudes whether calculated from RNA sequencing or RT-qPCR. There was no statistically significant difference in fold-change of expression measured by the two methods (all p-values>0.05, Student’s t-test).

### Neutrophil RNA expression discriminates IA from control groups

**Fig 3:**
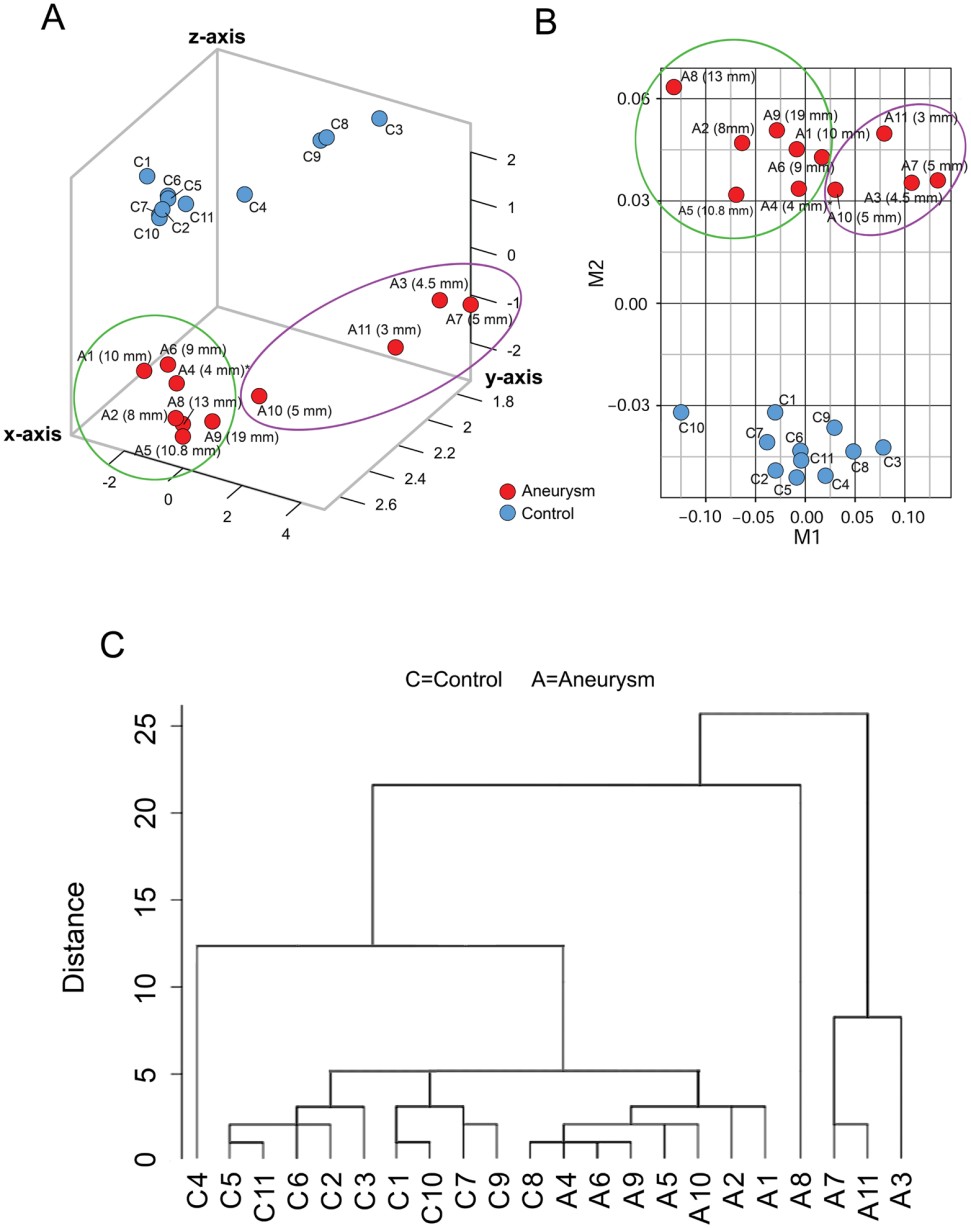
Dimensionality reduction analyses separate blood samples from IA patients and controls. (A) Principal component analysis (PCA) using all transcriptome data demonstrates aggregation of samples from IA patients (red) and controls (blue). Transcriptome data further separated samples from IA patients by aneurysm size, forming groups of large IAs (≥8 mm, with one exception) and small IAs (≤5 mm). (B) Multidimensional scaling (MDS) of transcriptome data further reduces dimensionality and mirrors the PCA results, also showing separation of IA (red) and control (blue) samples. (C) Hierarchical clustering using only the 82-transcript IA-associated signature also demonstrates separation of IA and control groups. Four aneurysms samples on the right were more distinct than others, while the rest of the samples segregated into three main clusters, two containing all control and one containing all IA (with one exception). Overall, 91% of the samples were grouped with their respective group.

To visualize how well neutrophil RNA expression differentiated aneurysm samples from control samples, we performed dimensionality reduction analyses by PCA and MDS using all neutrophil transcriptome data (13,377 transcripts). Fig 3A shows that the IA and control groups separated in the principal component space. Similarly, MDS also showed separation of patients with IAs and control subjects (Fig 3B). We also found that the transcriptome data segregated the patients with IAs by the size of each patient’s largest IA, forming two groups on both the PCA and MDS plots: large (≥8 mm, with one exception) and small (≤5 mm) (Fig 3A and 3B).

Using the expression signature of 82 transcripts (p<0.05 and absolute fold-change ≥2), we performed supervised hierarchical clustering to determine whether it could also discriminate patients with IAs from controls. On the dendrogram in Fig 3C, samples from IA and control groups are separated. Four samples from the IA group on the right were more distinct than the others. In the rest of the samples, one control (to the left) was separate, and all other samples segregated into 3 groups. Two groups contained all control samples and 1 group contained all IA samples (with 1 exception). In general, hierarchical clustering congregated 91% of the samples with their respective groups.

### Expression differences are consistent with leukocyte activation

To gain insight into the biology of neutrophil RNA expression differences we performed bioinformatics analyses using gene set enrichment analysis and physiological pathway modeling. Some tightly controlled pathways can be regulated by transcripts that show small but significant changes. To avoid missing potentially significant biological insights, we performed bioinformatics analysis using all 258 differentially expressed transcripts (p<0.05) regardless of fold-change. As detailed in Table 3, gene ontology analysis revealed that genes with higher neutrophil expression levels in the IA group were involved in defense response, leukocyte activation, stem cell maintenance, maintenance of cell number, cell activation, and stem cell development. Genes with lower expression levels in the IA group were involved in immune response and immune system process (Table 3).

**Table 3:** Gene ontology (GO) analysis*.

GO ID	Term	P-value	Q-value	Annotated Genes
***Genes Increased in IA***				
GO:0006952	Defense Response	3.13E-06	2.36E-03	*KLRC2*, *VNN1*, *C4BPA*, *CD300E*, *SH2D1B*, *CD247*, *GNLY*, *INHBB*, *CD1D*, *KIR2DS4*, *PRF1*, *ORM2*, *STAB1*, *FCER1A*, *CD86*
GO:0045321	Leukocyte Activation	8.23E-06	6.21E-03	*VNN1*, *CD1D*, *CD7*, *PRF1*, *FCER1A*, *SH2D1B*, *SOX4*, *CD86*, *CD247*
GO:0019827	Stem Cell Maintenance	8.44E-06	6.37E-03	*NOG*, *TCL1A*, *KLF10*, *SCT*, *SOX4*
GO:0098727	Maintenance of Cell Number	8.95E-06	6.76E-03	*NOG*, *TCL1A*, *KLF10*, *SCT*, *SOX4*
GO:0001775	Cell Activation	1.18E-05	8.92E-03	*VNN1*, *CD7*, *VWF*, *SH2D1B*, *SOX4*, *CD247*, *CD1D*, *PRF1*, *FCER1A*, *CD86*
GO:0048864	Stem Cell Development	1.20E-05	9.10E-03	*NOG*, *DAB2*, *TCL1A*, *KLF10*, *SCT*, *SOX4*
***Genes Decreased in IA***				
GO:0006955	Immune Response	1.17E-07	6.85E-05	*AIM2*, *LILRA4*, *FCRL5*, *IL8*, *MOV10*, *CYSLTR2*, *IFI35*, *PDCD1LG2*, *RGS1*, *CD274*, *CCL23*, *DDX60*, *OLFM4*, *GBP1*
GO:0002376	Immune System Process	3.32E-07	1.94E-04	*MOV10*, *CEBPE*, *SMPD3*, *IFI35*, *PDCD1LG2*, *CCL23*, *OLFM4*, *GBP1*, *AIM2*, *FCRL5*, *LILRA4*, *IL8*, *CYSLTR2*, *RGS1*, *CD274*, *DDX60*

* Gene set enrichment analysis was performed on significantly differentially expressed genes (p<0.05) in peripheral blood samples obtained from patients with intracranial aneurysms (IA). Significantly enriched ontologies present in genes with higher expression levels included defense response, leukocyte activation, stem cell maintenance, maintenance of cell number, cell activation, and stem cell development. Significantly enriched ontologies present in genes with lower expression levels included immune response and immune system process. Enriched ontologies from the GO database were considered significant at a false discovery rate-corrected p-value (q-value) <0.05.

**Fig 4:**
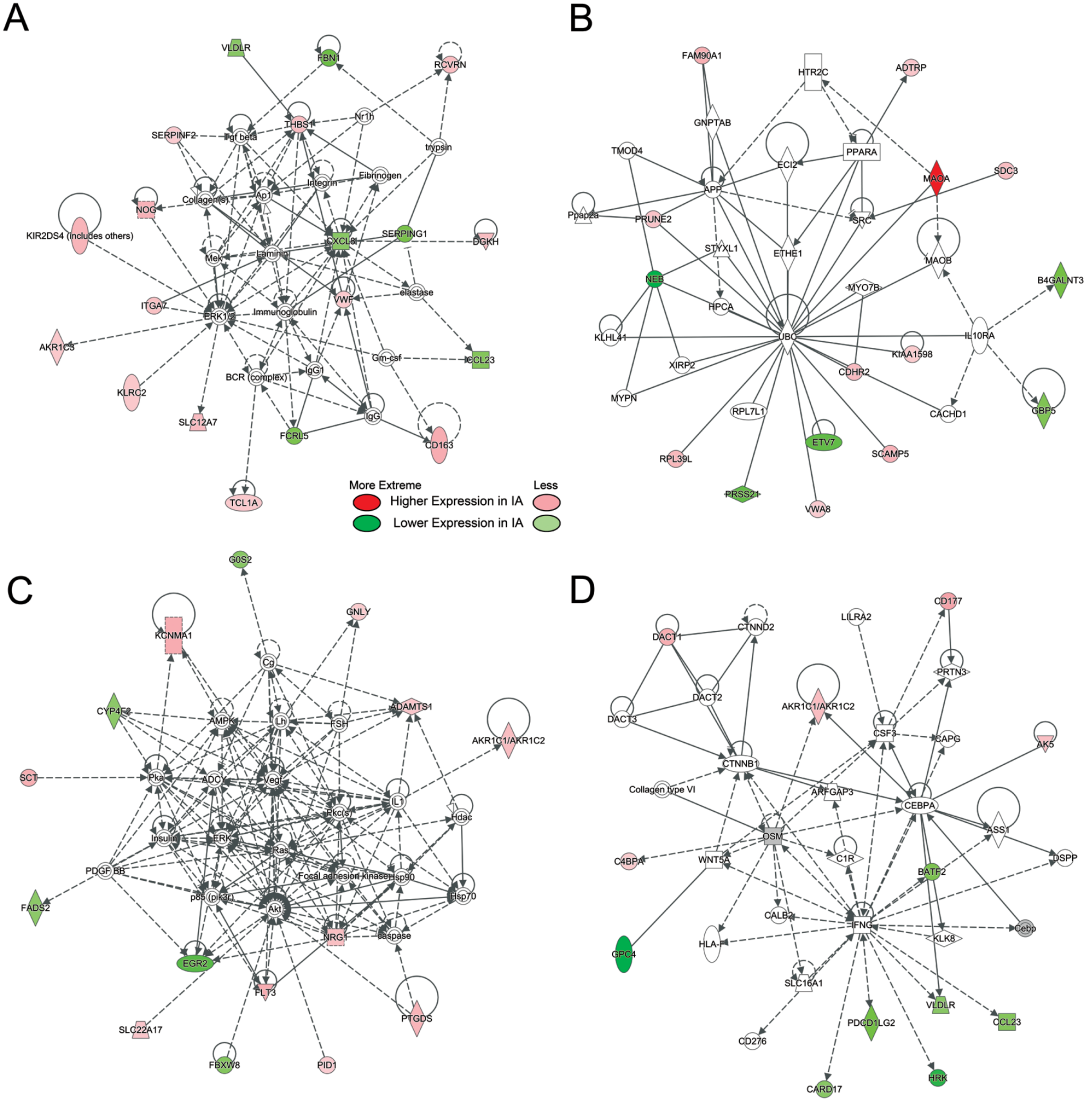
The 4 most regulated networks. Networks were derived from IPA of differentially expressed transcripts (p<0.05) in neutrophils from IA patients and controls. Transcripts with increased expression levels in patients with IAs are red; transcripts with lower expression levels in patients with IAs are green; and fold-change is represented by intensity. Non-differentially expressed transcripts with known interactions are not colored. (A) This network (p-score = 41) shows regulation of transcripts with increased expression by an *ERK1/2* and *AP1*. *IL8*, regulates transcripts with lower expression in samples from patients with IAs. (B) This network (p-score = 30) shows regulation of transcripts by *UBC*. (C) This network (p-score = 30) shows two nodes of regulation at *AKT* and *VEGF*. (D) This network (p-score = 23) shows regulation of transcripts with lower expression in IA samples by *IFNG*.

Physiological pathway modeling to identify networks of potential interactions revealed 4 networks with 7 signaling nodes forming hubs within the networks (Fig 4). These hubs included *ERK1/2* and *AP1*; *IL8* (*CXCL8*), *AKT* and *VEGF*; *UBC*; and *IFNG*. IPA indicated that these networks were consistent with activation of cellular movement and cardiovascular system function (network A), lipid metabolism (network B), cell-to-cell signaling and energy production (network C), and organismal injury, cell proliferation, and tissue morphology (network D). These functions are pertinent to neutrophil responses to intravascular perturbations.[27] See S5 Table for a list of names and biological functions of the transcripts in these 4 networks.

### Replication study in a new, unpaired population

**Fig 5:**
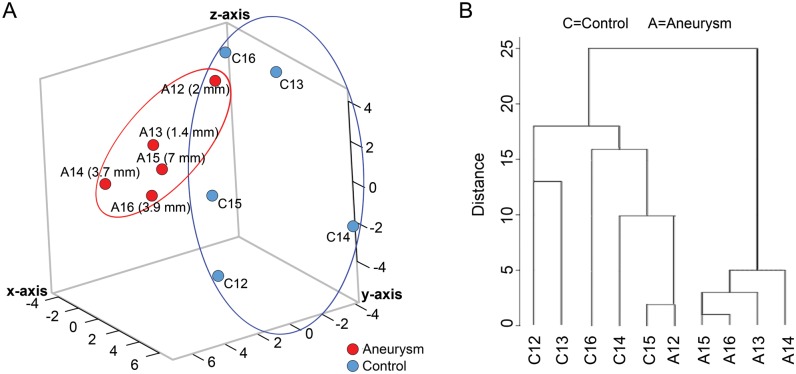
Replication study in a cohort of 10 new patients (5 with IAs). (A) Principal component analysis performed using the 82 IA-associated transcripts shows separation of IA from control samples in this unmatched cohort. (B) Hierarchical cluster analysis demonstrates separation of the IA and control samples, with the exception of one IA sample that was grouped with controls.

To determine whether expression of the IA-associated signature could separate patients with IAs from controls in an independent cohort, we performed a small replication study. We recruited 10 additional patients (5 with IA, 5 IA-free controls) but did not control for demographics and comorbidities in order to assess the signature’s potential for segregating patients in heterogeneous populations (see S6 Table for clinical characteristics). Patients with IAs had aneurysms ranging in size from 1.4–7 mm and included one individual with multiple aneurysms (S7 Table). From these patients’ peripheral blood samples, we isolated neutrophils and extracted neutrophil RNA and performed next-generation RNA sequencing to obtain FPKM levels of the 82 IA-associated transcripts. To visualize how these transcripts could distinguish the IA group from the control group, we performed PCA and hierarchical clustering. With the exception of one IA sample, PCA demonstrated separation of the two groups in the principal component space (Fig 5A). Hierarchical clustering mirrored this result, grouping the IA and control samples separately, with the exception of one IA sample (Fig 5B).

### Availability of RNA expression data

Raw RNA sequencing data files and processed transcript expression levels for the experiments described in this publication can be found at NCBI’s GEO (accession no. GSE106520). Deidentified patient metadata is presented in S8 Table and can also be found at NCBI’s GEO with the expression data.

## Discussion

We performed transcriptome profiling on circulating neutrophils from paired patients with and without IAs and identified an aneurysm-associated signature of 82 transcripts. These transcripts discriminated patients with and without IA in hierarchical cluster analysis. In a replication study, this signature also distinguished patients with IAs from controls in an unpaired cohort. These findings present the exciting potential for developing predictive biomarkers that use this signature to identify patients with IAs.

### Previous efforts in search of circulating aneurysm biomarkers

The search for circulating biomarkers for unruptured IAs has spanned more than two decades. A meta-analysis[28] of IA biomarker publications from 1994–2015 found 5 studies that linked IA presence to specific biomolecules in the blood. These studies found that serum elastase-to-A1AT ratios [29] and LPA [30], VEGF [31], MCP-1, IL-1β, TNF-α [32], and GM-CSF levels [33] were elevated in patients with unruptured aneurysms. However, in the present study, we did not observe significantly higher mRNA levels for these proteins in neutrophils from patients with IA. This may be because these proteins originate from sources other than neutrophils or may not be sufficiently unique to IA to be identified by our analysis.

One common trait of the previously-identified potential proteins markers, is that they are ubiquitous, being involved in a wide range of physiological and pathological functions. Thus, in addition to IA, they may also signify various vascular diseases. For example, serum VEGF is also increased during peripheral artery stenosis [34], plasma MCP-1 is also elevated in thromboembolic hypertension [35], and LPA is elevated in plasma of patients with vascular dementia [36]. Perhaps for this reason, significant follow-up efforts have not been made towards subsequent biomarker development and validation on the basis of these studies.

An alternative approach to identifying potential biomarkers is to profile the transcriptome of the circulating blood, which affords screening for multitudes of potential markers and can provide insight into novel disease mechanisms that may be specific to IA. Recently, circulating RNA expression signatures of unruptured IAs were found in microarray studies. In IA patients, Jin et al. [37] found 77 differentially expressed plasma microRNAs that were involved in proliferation, apoptosis, molecular activation, transport, and differentiation; Li et al. [38] discovered 119 differentially expressed plasma microRNAs related to inflammatory responses and connective tissue disorders; and Sabatino et al. [39] identified 53 differentially expressed mRNAs from peripheral blood mononuclear cells that were related to increased cell proliferation and apoptosis. These findings indicate that IA is associated with altered expression of a large number of transcripts from various circulating blood components. Each study benefited from the use of high-throughput technology (i.e. microarray). We believe that such technologies open the door to discovery of IA-specific signatures consisting of many transcripts.

On the basis of this vision, we conducted the current study, albeit using next-generation RNA sequencing. This latest high-throughput technology affords two key advantages over microarrays used in previous investigations: (1) it offers a larger dynamic range, facilitating detection of expression differences in low-abundance transcripts; and (2) it avoids predetermined probes, allowing examination of novel RNAs (i.e., splice variants, non-coding RNAs, gene isoforms) [40]. These capabilities led us to discover a signature of 82 transcripts, containing several uncharacterized and/or non-coding RNAs, which cannot be detected on conventional microarrays. They include *C21orf15*, *LOC100131289*, *FLJ27354*, *LOC100507387*, *LINC00482*, *C1orf226*, and *LOC730441*. To our knowledge, these novel transcripts have not been associated with any other diseases. Further research is required to determine their functions and how they contribute to IA pathophysiology.

We also designed our study to avoid common pitfalls of expression profiling studies [37–39]. First, to avoid misclassification, we used DSA to confirm that the control subjects did not have aneurysms. Previous studies did not perform such imaging. Second, to find RNA expression differences due to the presence of an IA and not confounding factors, we paired the subjects by demographics and comorbidities. Previous studies typically used healthy subjects or spouses as controls. Third, we performed a replication study in an independent, unpaired cohort to investigate whether the signature can distinguish patients with IA in a general population. These measures helped to increase the likelihood that the discovered signature is associated with IA presence.

### Circulating neutrophils and intracranial aneurysms

Intracranial aneurysm natural history is characterized by mounting inflammatory responses and progressive degradation of the aneurysmal wall, starting from initial pro-inflammatory changes in smooth muscle cells that lead to overproduction of matrix metalloproteinases (MMPs) [5, 41]. Once the aneurysmal pouch is formed, it harbors a hemodynamic environment conducive to macrophage and neutrophil infiltration into the wall, which is aided by a local increase of plasma chemokines and cytokines (IL-1β, IL-17, TNF-α) in the lumen [11, 42]. These inflammatory infiltrates massively produce MMPs to further degrade the aneurysm wall and advance its growth and rupture [5, 43]. This is evidenced by gene expression studies of human aneurysmal tissues, which found increased matrix degradation processes, inflammatory processes, and production of inflammatory cytokines and chemoattractant proteins in the IA wall [8, 9]. Furthermore, Yu et al. found that differences in DNA methylation in aneurysmal tissue act to promote inflammatory signaling through the NF-KB, JNK-STAT, and ERK/JNK pathways [44], uncovering a potential epigenetic underpinning to dysregulated inflammation during IA.

The role of neutrophils in IA pathophysiology may be complex and is not well understood. Besides secreting MMP-9, activated neutrophils also release NGAL and MPO, which indirectly contribute to extracellular matrix degradation and cytotoxicity, respectively. Increased NGAL in aneurysm tissue modulates the activity of MMP-9, protecting it from degradation and thus aiding aneurysm progression [11]. Increased MPO, an inflammatory enzyme, elicits oxidative stress and pro-inflammatory cell signaling through production of reactive oxygen species [10]. It has been observed that plasma levels of NGAL and MPO are increased in the blood of patients with aneurysms [11, 45]. Furthermore, both of these proteins can have autocrine effects that promote neutrophil activation [46, 47], which could lead to expression changes observed in our study. Interestingly, we found significantly increased expression of *SLC22A17*, which is the NGAL receptor, in neutrophils from patients with IAs. This may reflect a possible interaction with circulating NGAL. However, we did not observe significantly higher levels of *NGAL* or *MPO* in circulating neutrophils, suggesting that these proteins may originate from the aneurysm sac itself, or other circulating cells.

Further analysis of our expression data supports an association between activated circulating neutrophils and IA presence. Gene set enrichment analysis reveals that neutrophils from IA patients have higher levels of gene expression associated with leukocyte activation. This is evidenced by increased expression levels of several CD antigens from the “leukocyte activation” ontology (*CD1D*, *CD7*, *CD86*, and *CD247*) as well as *CD177*, a marker of neutrophil activation. IPA also reveals functions indicative of activated neutrophils, showing networks consistent with activation of cellular movement, cell-to-cell signaling, and cell proliferation. The fact that neutrophil expression data segregated aneurysms by size in PCA and MDS (Fig 3A and 3B) may indicate a correlation between the degree of IA advancement and neutrophil activation. Overall, our findings suggest that peripheral neutrophil activation may play a role in IA development.

### Limitations

This proof-of-concept study has three main limitations. First, our IA-associated expression signature was extracted from a small sample. However, by selecting transcripts with large effect size (fold-change ≥2), performing confirmatory qPCR, and demonstrating separation of IAs from controls in an independent cohort, we were able to increase confidence in the discovered signature. We hope this exploratory effort lays a foundation for future studies in larger cohorts with increased statistical power. Second, all of our study subjects were recruited from patients receiving cerebral imaging at our center; thus, there may be a potential for selection bias. Limiting subjects to those receiving imaging was necessary in our study to confirm the presence or absence of IA, but using broader, randomized patient populations from multiple centers would have been beneficial. Lastly, despite patient matching between the groups to remove confounding factors (potentially contributing to the IA signature), there is a possibility that the found differential neutrophil expression in the signature could be caused by other conditions. It is known that neutrophil activation can also occur in other vascular pathologies and inflammatory states. Further research is needed to investigate the specificity of this IA-associated signature to IA.

## Conclusions

Early IA detection is important for preventing rupture; therefore, blood-based diagnostics could change the landscape of IA management. In this preliminary, exploratory effort, we identified an IA-associated RNA expression signature of 82 transcripts in circulating neutrophils. Despite our small sample size, this signature demonstrated a statistical power >0.80 and was able to distinguish patients with IAs from paired controls in several analyses. These transcripts also separated patients with IAs from unpaired controls in a small population. These findings need to be validated in larger, more diverse cohorts.

## Supporting information

S1 Fig The patient medical history form. This form in the patient’s medical record was evaluated to retrieve the patient’s clinical information.
(TIF)

S2 Fig White blood cell populations in the IA and control groups. There was no significant difference in white blood cell count or leukocyte ratios between patients with IAs (n = 11) and controls (n = 7, no data were available for 4 of the controls). (A) Complete blood count data recorded within 3 months of blood collection showed no significant difference between-groups in the concentrations of leukocytes, erythrocytes, platelets, neutrophils, lymphocytes, or monocytes (p>0.05, Student’s t-test). (B) There was also no significant difference in the percentage (%) per 100 leukocytes for neutrophils, lymphocytes, monocytes, eosinophils, and basophils between patients with and without IA (p>0.05, Student’s t-test). (Data points = average values, error bars = standard error).
(TIF)

S1 Table Primers used for qPCR and their efficiencies*. *Primers were selected using Primer3 and NCBI’s Primer Blast. All efficiencies were within the range of 0.90–1.10. (bp = base pair, Eff. = efficiency, Prod. = product, Temp. = temperature).
(DOCX)

S2 Table RNA quality*. *The quality of the RNA samples was assessed by the 260/280 ratio and the RIN. (RIN = RNA integrity number).
(DOCX)

S3 Table Characteristics of 16 intracranial aneurysms in the group of 11 patients with IAs (3 patients had multiple intracranial aneurysms)*. *Aneurysm size ranged from 1.5mm to 19mm. Ten of 16 IAs (63%) were classified as small (greatest diameter <7mm) and 6 (37%) were classified as large (greatest diameter ≥7 mm). The aneurysms were situated at various locations in the Circle of Willis, with most being around the internal carotid artery (ICA) and its branches. Two patients with IAs had a family history of the disease. In general, digital subtraction angiography was performed for either confirmation of IA presence after an incidental finding of IA on noninvasive imaging, or for follow-up imaging of a previously detected IA. (ACA = anterior cerebral artery, AComA = anterior communicating artery, BT = basilar terminus, CT = computed tomography, DSA = digital subtraction angiography, IA = intracranial aneurysm, ICA = internal carotid artery, MCA = middle cerebral artery, MRA = magnetic resonance angiography, MRI = magnetic resonance imaging, PComA = posterior communicating artery, VB = vertebrobasilar).
(DOCX)

S4 Table RNA Sequencing Quality Control Analysis*. *The quality of the RNA sequencing experiments was measure pre-alignment via FASTQC and post-alignment via MultiQC. Overall, prior to alignment all samples had an average of 53.75 M sequences. MultiQC reported that the sequencing experiments had an average of 49.09 M mapped reads with a 96.13% read mapping rate, and detected an average of 17259 transcripts (transcripts with FPKM>0). (Align. = alignment, M. = million, Seqs. = sequences, Qual. = quality).
(DOCX)

S5 Table Transcripts involved in the 4 networks constructed by Ingenuity Pathway Analysis (IPA)*. *A table of the names of transcripts included in the top 4 networks derived from IPA, as well as the top diseases and functions of these transcripts. Neutrophil transcripts in bold were differentially expressed between patients with and without IA (p-value<0.05). Each network’s p-score was derived from its p-value [p-score = -Log10 (p-value)] calculated by the Fisher’s exact test. For a network with a p-score of 10, the odds of generating this network by chance alone is less than 1 out of 10^10^.
(DOCX)

S6 Table Clinical characteristics of the unpaired cohort of 5 patients with intracranial aneurysms and 5 control subjects without intracranial aneurysms (confirmed on imaging)*. *(IA = intracranial aneurysm, SE = standard error, Q = quartile).
(DOCX)

S7 Table Characteristics of 6 intracranial aneurysms in the replication group of 5 patients with IAs (one patients had multiple intracranial aneurysms)*. *Aneurysm size ranged from 3.5 mm to 7 mm. Five of 6 IAs (83%) were classified as small (greatest diameter <7mm) and 1 (17%) was classified as large (greatest diameter ≥7 mm). The aneurysms were situated at various locations in the Circle of Willis, with most being in the anterior vasculature (ACA and MCA). (ACA = anterior cerebral artery, AComA = anterior communicating artery, BT = basilar terminus, CT = computed tomography, DSA = digital subtraction angiography, IA, intracranial aneurysm, MCA = middle cerebral artery, MRA = magnetic resonance angiography, MRI = magnetic resonance imaging).
(DOCX)

S8 Table Deidentified patient metadata*. *(M = male, F = female, Y = yes, N = no, HT = hypertension, HL = hyperlipidemia, CAD = coronary artery disease, S Hx = stroke history, DM = diabetes mellitus, OA = osteoarthritis).
(DOCX)
