# Supplementary figures and images for: Circulating neutrophil transcriptome may reveal intracranial aneurysm signature
Source: PLoS One. 2018 Jan 17;13(1):e0191407. doi: 10.1371/journal.pone.0191407 (PMC5771622; doi:10.1371/journal.pone.0191407)

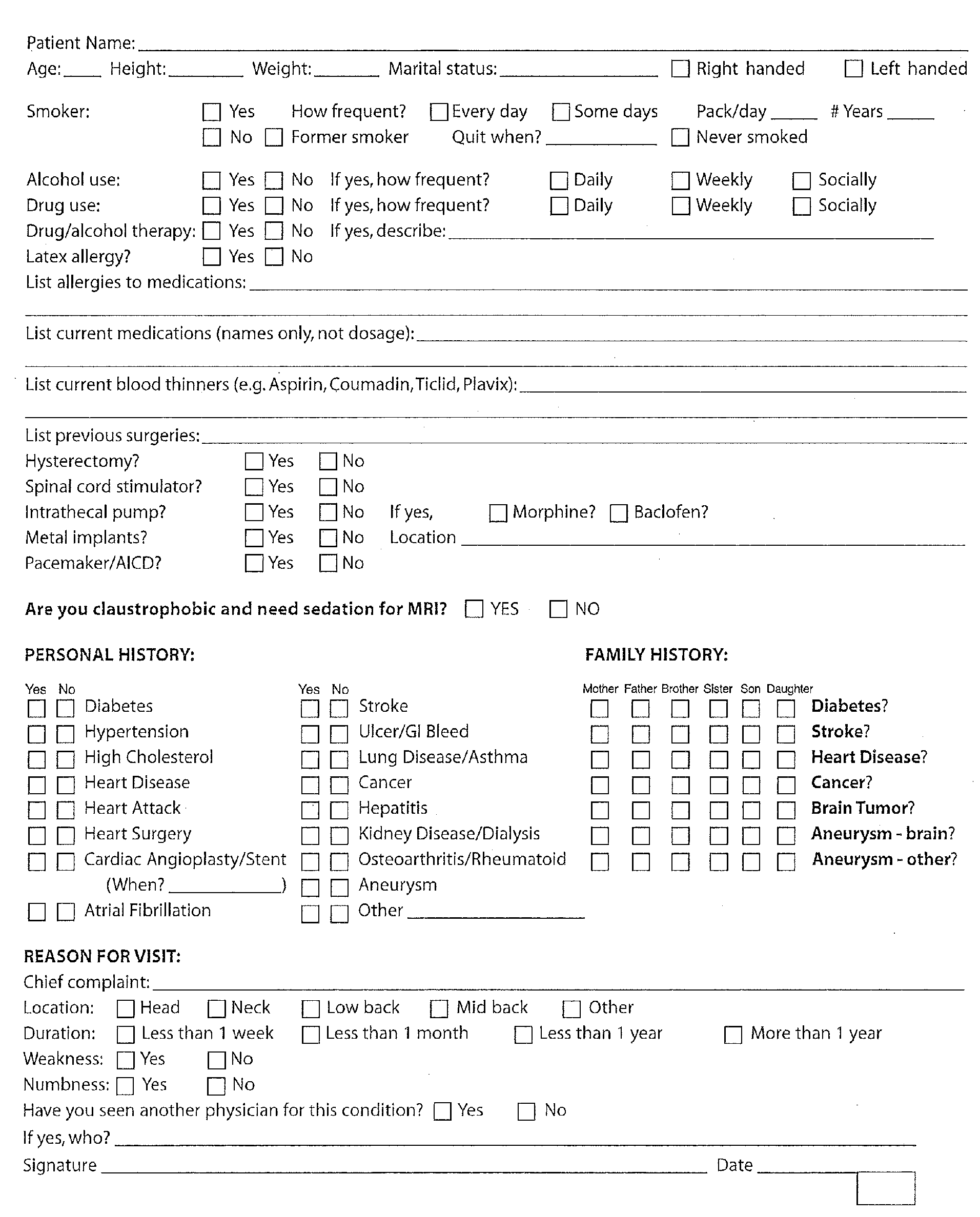

Supplement: S1 Fig — This form in the patient’s medical record was evaluated to retrieve the patient’s clinical information. (TIF) [file pone.0191407.s001.tif]

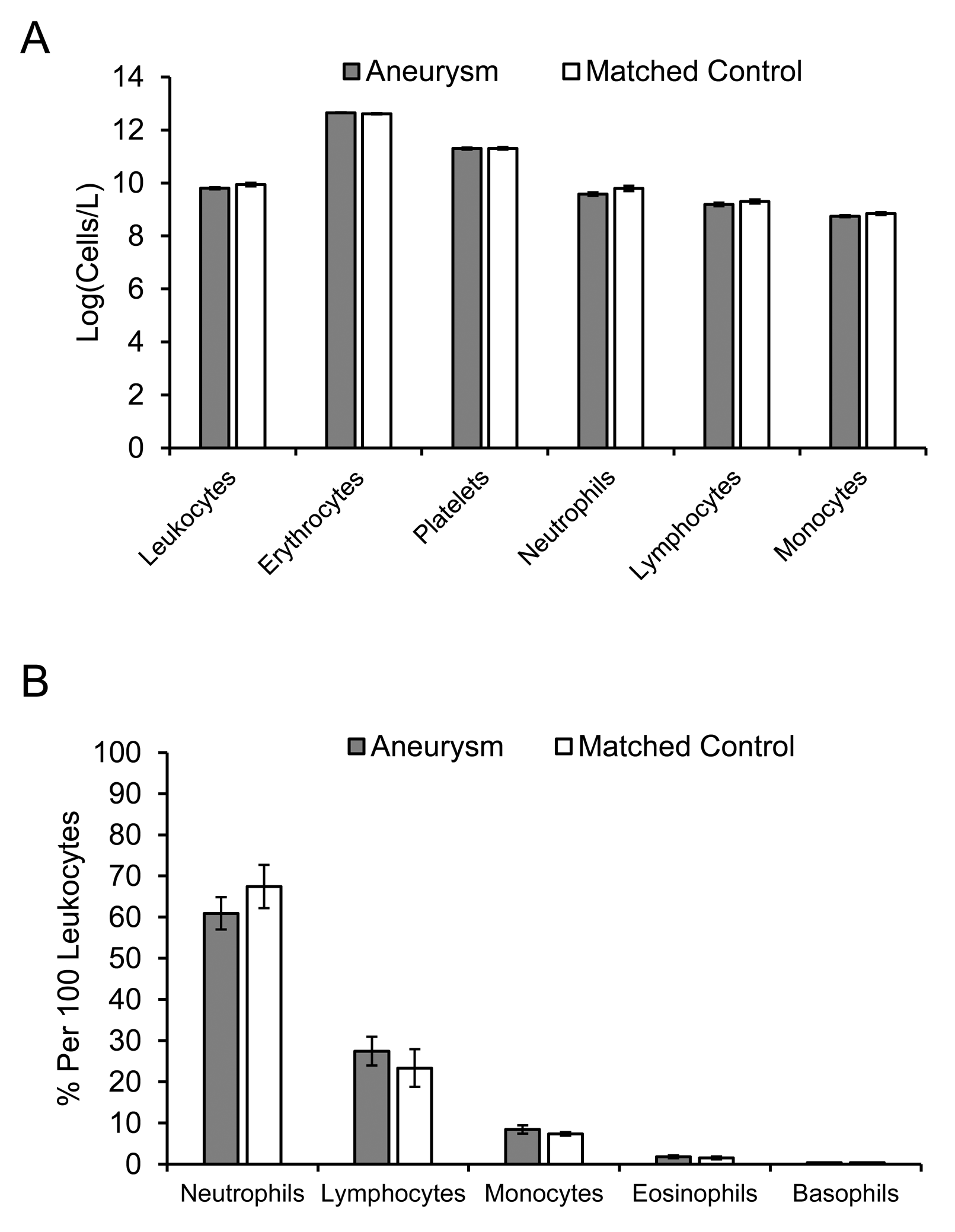

Supplement: S2 Fig — There was no significant difference in white blood cell count or leukocyte ratios between patients with IAs (n = 11) and controls (n = 7, no data were available for 4 of the controls). (A) Complete blood count data recorded within 3 months of blood collection showed no significant difference between-groups in the concentrations of leukocytes, erythrocytes, platelets, neutrophils, lymphocytes, or monocytes (p>0.05, Student’s t-test). (B) There was also no significant difference in the percentage (%) per 100 leukocytes for neutrophils, lymphocytes, monocytes, eosinophils, and basophils between patients with and without IA (p>0.05, Student’s t-test). (Data points = average values, error bars = standard error). (TIF) [file pone.0191407.s002.tif]
