# Supplementary material for: Circulating neutrophil transcriptome may reveal intracranial aneurysm signature
Source: PLoS One. 2018 Jan 17;13(1):e0191407. doi: 10.1371/journal.pone.0191407 (PMC5771622; doi:10.1371/journal.pone.0191407)
Supplement: S1 Table — *Primers were selected using Primer3 and NCBI’s Primer Blast. All efficiencies were within the range of 0.90–1.10. (bp = base pair, Eff. = efficiency, Prod. = product, Temp. = temperature). (DOCX) [file pone.0191407.s003.docx]

**S1 Table. Primers used for qPCR and their efficiencies.***

| **Transcript** | **Primer Sequence** | **Annealing**  **Temp. (^o^C)** | **Eff.** | **PCR Prod.**  **Length (bp)** |
| --- | --- | --- | --- | --- |
| *CD177* | 5'-ACACACGGAAACTTGGCTCA-3' | 60.0 | 1.04 | 124 |
|  | 5'-CCAGGGTTGATGTGAGTCCTAC-3' |  |  |  |
| *NAAA* | 5'-AACTTCGAAGCAGCTGTTGG-3' | 60.0 | 1.01 | 195 |
|  | 5'-TGGCTTCCAGTGGTCGTAAT-3' |  |  |  |
| *SERPING1* | 5'-AGATCTTCCACAGCCCAGAC-3' | 60.0 | 0.94 | 104 |
|  | 5'-GGCGTCACTGTTGTTGCTTA-3' |  |  |  |
| *GBP5* | 5'-TTGGGCATCACTCAGGCTAA-3' | 60.0 | 1.04 | 93 |
|  | 5'-CCCAGTTGAAAGCTGCACAT-3' |  |  |  |
| *IL8* | 5'-CAGAGACAGCAGAGCACACA-3' | 60.0 | 1.07 | 70 |
|  | 5'-GTGAGATGGTTCCTTCCGGT-3' |  |  |  |
| *GAPDH* | 5'-CGCTCTCTGCTCCTCCTGTT-3' | 60.0 | 1.09 | 81 |
|  | 5'-CCATGGTGTCTGAGCGATGT-3' |  |  |  |

*Primers were selected using Primer3 and NCBI’s Primer Blast. All efficiencies were within the range of 0.90–1.10. (bp=base pair, Eff.=efficiency, Prod.=product, Temp.=temperature)
