# Supplementary material for: Circulating neutrophil transcriptome may reveal intracranial aneurysm signature
Source: PLoS One. 2018 Jan 17;13(1):e0191407. doi: 10.1371/journal.pone.0191407 (PMC5771622; doi:10.1371/journal.pone.0191407)
Supplement: S2 Table — *The quality of the RNA samples was assessed by the 260/280 ratio and the RIN. (RIN = RNA integrity number). (DOCX) [file pone.0191407.s004.docx]

**S2 Table. RNA Quality.***

|  | **ID** | **Class** | **260/280** | **RIN** | |  |
| --- | --- | --- | --- | --- | --- | --- |
| ***Discovery Cohort*** | | | | | |  |
|  | C1 | Control | 2.07 | | 7.0 |  |
|  | C2 | Control | 2.02 | | 7.4 |  |
|  | C3 | Control | 2.05 | | 6.6 |  |
|  | C4 | Control | 1.92 | | 6.5 |  |
|  | C5 | Control | 1.99 | | 7.1 |  |
|  | C6 | Control | 2.08 | | 7.1 |  |
|  | C7 | Control | 2.05 | | 7.9 |  |
|  | C8 | Control | 1.96 | | 6.7 |  |
|  | C9 | Control | 2.08 | | 6.2 |  |
|  | C10 | Control | 2.04 | | 7.3 |  |
|  | C11 | Control | 1.97 | | 6.4 |  |
|  | A1 | Aneurysm | 2.04 | | 7.8 |  |
|  | A2 | Aneurysm | 2.07 | | 7.5 |  |
|  | A3 | Aneurysm | 2.06 | | 8.1 |  |
|  | A4 | Aneurysm | 2.02 | | 6.1 |  |
|  | A5 | Aneurysm | 2.03 | | 7.3 |  |
|  | A6 | Aneurysm | 1.99 | | 6.5 |  |
|  | A7 | Aneurysm | 2.02 | | 7.5 |  |
|  | A8 | Aneurysm | 2.05 | | 7.7 |  |
|  | A9 | Aneurysm | 2.07 | | 6.0 |  |
|  | A10 | Aneurysm | 1.95 | | 7.2 |  |
|  | A11 | Aneurysm | 1.97 | | 6.9 |  |
| ***Replication Cohort*** | | | | | |  |
|  | C12 | Control | 2.03 | | 6.0 |  |
|  | C13 | Control | 2.04 | | 6.6 |  |
|  | C14 | Control | 2.05 | | 6.0 |  |
|  | C15 | Control | 2.02 | | 8.2 |  |
|  | C16 | Control | 2.04 | | 6.0 |  |
|  | A12 | Aneurysm | 2.06 | | 6.0 |  |
|  | A13 | Aneurysm | 1.97 | | 6.4 |  |
|  | A14 | Aneurysm | 2.00 | | 6.9 |  |
|  | A15 | Aneurysm | 1.96 | | 7.4 |  |
|  | A16 | Aneurysm | 2.03 | | 7.2 |  |

* The quality of the RNA samples was assessed by the 260/280 ratio and the RIN. (RIN=RNA integrity number)
