# Supplementary material for: Circulating neutrophil transcriptome may reveal intracranial aneurysm signature
Source: PLoS One. 2018 Jan 17;13(1):e0191407. doi: 10.1371/journal.pone.0191407 (PMC5771622; doi:10.1371/journal.pone.0191407)
Supplement: S3 Table — *Aneurysm size ranged from 1.5mm to 19mm. Ten of 16 IAs (63%) were classified as small (greatest diameter <7mm) and 6 (37%) were classified as large (greatest diameter ≥7 mm). The aneurysms were situated at various locations in the Circle of Willis, with most being around the internal carotid artery (ICA) and its branches. Two patients with IAs had a family history of the disease. In general, digital subtraction angiography was performed for either confirmation of IA presence after an incidental finding of IA on noninvasive imaging, or for follow-up imaging of a previously detected IA. (ACA = anterior cerebral artery, AComA = anterior communicating artery, BT = basilar terminus, CT = computed tomography, DSA = digital subtraction angiography, IA = intracranial aneurysm, ICA = internal carotid artery, MCA = middle cerebral artery, MRA = magnetic resonance angiography, MRI = magnetic resonance imaging, PComA = posterior communicating artery, VB = vertebrobasilar). (DOCX) [file pone.0191407.s005.docx]

**S3 Table**. **Characteristics of 16 intracranial aneurysms in the group of 11 patients with IAs (3 patients had multiple intracranial aneurysms)***

| **ID** | **IA**  **Size**  **(mm)** | **IA**  **Location** | **Presence of**  **Additional IAs** | **Family**  **History of IA** | **Indications for DSA** |
| --- | --- | --- | --- | --- | --- |
| A1 | 10 | VB junction | No | No | MRI for hand numbness indicated possible IA |
| A2 | 8 | Ophthalmic | No | No | Follow-up imaging of known IA |
| A3 | 4.5 | MCA | No | No | Incidental finding on CT indicated possible IA |
| A4 | 4 | Ophthalmic | Yes: +2 (1.5 mm and 3 mm ICA) | No | MRI for headache indicated possible IA |
| A5 | 10.8 | MCA | Yes: +2 (2.3 mm MCA, small AComA) | No | Incidental finding on MRI indicated possible IA |
| A6 | 9 | PComA | No | No | Follow-up of known IA |
| A7 | 5 | BT | No | No | MRA and CT for tremor revealed possible IA |
| A8 | 13 | ACA | No | Yes | MRI for decreased vision in left eye indicated possible IA |
| A9 | 19 | ICA | No | No | MRI for double vision indicated possible IA |
| A10 | 5 | ICA | Yes: +1 (3.5 mm ICA) | Yes | MRI for tremors indicated possible IA |
| A11 | 3 | BT | No | No | MRI for headache indicated possible IA |

*Aneurysm size ranged from 1.5mm to 19mm. Ten of 16 IAs (63%) were classified as small (greatest diameter <7mm) and 6 (37%) were classified as large (greatest diameter ≥7 mm). The aneurysms were situated at various locations in the Circle of Willis, with most being around the internal carotid artery (ICA) and its branches. Two patients with IAs had a family history of the disease. In general, digital subtraction angiography was performed for either confirmation of IA presence after an incidental finding of IA on noninvasive imaging, or for follow-up imaging of a previously detected IA. (ACA=anterior cerebral artery, AComA=anterior communicating artery, BT=basilar terminus, CT=computed tomography, DSA=digital subtraction angiography, IA=intracranial aneurysm, ICA=internal carotid artery, MCA=middle cerebral artery, MRA=magnetic resonance angiography, MRI=magnetic resonance imaging, PComA=posterior communicating artery, VB=vertebrobasilar)
