# Supplementary material for: Circulating neutrophil transcriptome may reveal intracranial aneurysm signature
Source: PLoS One. 2018 Jan 17;13(1):e0191407. doi: 10.1371/journal.pone.0191407 (PMC5771622; doi:10.1371/journal.pone.0191407)
Supplement: S4 Table — *The quality of the RNA sequencing experiments was measure pre-alignment via FASTQC and post-alignment via MultiQC. Overall, prior to alignment all samples had an average of 53.75 M sequences. MultiQC reported that the sequencing experiments had an average of 49.09 M mapped reads with a 96.13% read mapping rate, and detected an average of 17259 transcripts (transcripts with FPKM>0). (Align. = alignment, M. = million, Seqs. = sequences, Qual. = quality). (DOCX) [file pone.0191407.s006.docx]

**S4 Table. RNA Sequencing Quality Control Analysis.***

|  |  |  | **FASTQC (Pre-Align.)** | | | | **MultiQC (Post-Align.)** | |  |  |
| --- | --- | --- | --- | --- | --- | --- | --- | --- | --- | --- |
|  | **ID** | **Class** | **M. Seqs.** | **Poor Qual. Seqs.** | **Seqs. Length** | **% GC** | **% Aligned** | **M. Aligned** | | **Detected Transcripts** |
| ***Discovery Cohort*** | | | |  | |  | |  | |  |
|  | C1 | Control | 27 | 0 | 51 | 48 | 96.50 | 24.8 | | 17050 |
|  | C2 | Control | 59.3 | 0 | 51 | 49 | 96.50 | 54.4 | | 17930 |
|  | C3 | Control | 35.9 | 0 | 51 | 49 | 94.90 | 32.3 | | 18291 |
|  | C4 | Control | 68.9 | 0 | 51 | 49 | 96.60 | 63.3 | | 18465 |
|  | C5 | Control | 53.3 | 0 | 51 | 50 | 95.80 | 48.5 | | 17961 |
|  | C6 | Control | 80.3 | 0 | 51 | 50 | 96.30 | 73.5 | | 18418 |
|  | C7 | Control | 97.4 | 0 | 51 | 50 | 96.00 | 88.9 | | 18066 |
|  | C8 | Control | 67.8 | 0 | 51 | 49 | 96.40 | 62.2 | | 18714 |
|  | C9 | Control | 36.2 | 0 | 51 | 49 | 96.10 | 33.2 | | 17966 |
|  | C10 | Control | 79.3 | 0 | 51 | 49 | 96.80 | 72.7 | | 17366 |
|  | C11 | Control | 89.1 | 0 | 51 | 50 | 95.70 | 81.1 | | 18479 |
|  | A1 | Aneurysm | 48.1 | 0 | 51 | 49 | 96.90 | 44.5 | | 18003 |
|  | A2 | Aneurysm | 35.7 | 0 | 51 | 48 | 96.80 | 32.9 | | 17223 |
|  | A3 | Aneurysm | 60.4 | 0 | 51 | 49 | 96.90 | 55.9 | | 18662 |
|  | A4 | Aneurysm | 55.9 | 0 | 51 | 49 | 96.90 | 51.4 | | 18155 |
|  | A5 | Aneurysm | 61.3 | 0 | 51 | 49 | 97.20 | 56.9 | | 17682 |
|  | A6 | Aneurysm | 23.4 | 0 | 51 | 49 | 95.90 | 21.4 | | 17366 |
|  | A7 | Aneurysm | 35.8 | 0 | 51 | 49 | 96.40 | 32.3 | | 18625 |
|  | A8 | Aneurysm | 26.9 | 0 | 51 | 50 | 97.10 | 24.9 | | 16401 |
|  | A9 | Aneurysm | 29.1 | 0 | 51 | 50 | 96.60 | 26.8 | | 17311 |
|  | A10 | Aneurysm | 59.5 | 0 | 51 | 49 | 96.00 | 54.1 | | 18617 |
|  | A11 | Aneurysm | 14.6 | 0 | 51 | 49 | 95.00 | 13 | | 17401 |
| ***Replication Cohort*** | | | |  | |  | |  | |  |
|  | C12 | Control | 74.4 | 0 | 51 | 50 | 95.10 | 66.8 | | 18329 |
|  | C13 | Control | 66 | 0 | 51 | 50 | 95.50 | 60 | | 18579 |
|  | C14 | Control | 58.5 | 0 | 51 | 51 | 95.50 | 53 | | 18273 |
|  | C15 | Control | 64.3 | 0 | 51 | 51 | 94.90 | 57.9 | | 18448 |
|  | C16 | Control | 47.3 | 0 | 51 | 50 | 95.30 | 42.7 | | 18039 |
|  | A12 | Aneurysm | 42.2 | 0 | 51 | 51 | 95.50 | 38.3 | | 17697 |
|  | A13 | Aneurysm | 75.3 | 0 | 51 | 51 | 95.90 | 68.6 | | 18313 |
|  | A14 | Aneurysm | 64.9 | 0 | 51 | 50 | 96.60 | 59.5 | | 17546 |
|  | A15 | Aneurysm | 39.8 | 0 | 51 | 50 | 97.40 | 36.8 | | 18031 |
|  | A16 | Aneurysm | 42.1 | 0 | 51 | 50 | 95.20 | 38.2 | | 17330 |

* The quality of the RNA sequencing experiments was measure pre-alignment via FASTQC and post-alignment via MultiQC. Overall, prior to alignment all samples had an average of 53.75 M sequences. MultiQC reported that the sequencing experiments had an average of 49.09 M mapped reads with a 96.13% read mapping rate, and detected an average of 17259 transcripts (transcripts with FPKM>0). (Align.=alignment, M.=million, Seqs.=sequences, Qual.=quality)
