# Supplementary material for: Circulating neutrophil transcriptome may reveal intracranial aneurysm signature
Source: PLoS One. 2018 Jan 17;13(1):e0191407. doi: 10.1371/journal.pone.0191407 (PMC5771622; doi:10.1371/journal.pone.0191407)
Supplement: S5 Table — *A table of the names of transcripts included in the top 4 networks derived from IPA, as well as the top diseases and functions of these transcripts. Neutrophil transcripts in bold were differentially expressed between patients with and without IA (p-value<0.05). Each network’s p-score was derived from its p-value [p-score = -Log10 (p-value)] calculated by the Fisher’s exact test. For a network with a p-score of 10, the odds of generating this network by chance alone is less than 1 out of 1010. (DOCX) [file pone.0191407.s007.docx]

**S5 Table. Transcripts involved in the 4 networks constructed by Ingenuity Pathway Analysis (IPA).***

| **Network** | **Molecules in Network** | **P-Score** | **Focus Molecules** | **Top Diseases and Functions** |
| --- | --- | --- | --- | --- |
| **A** | **AKR1C3**, Ap1, BCR (complex), **CCL23**, **CD163**, **CXCL8**, Collagen(s), **DGKH**, ERK1/2, **FBN1**, **FCRL5**, Fibrinogen, Gm-csf, **ITGA7**, IgG, IgG1, Immunoglobulin, Integrin, **KIR2DS4** (includes others), **KLRC2**, Laminin, Mek, **NOG**, Nr1h, **RCVRN**, **SERPINF2**, **SERPING1**, **SLC12A7**, **TCL1A**, **THBS1**, Tgf beta, **VLDLR**, **VWF**, elastase, trypsin | 41 | 19 | Carbohydrate Metabolism, Cardiovascular System Development and Function, Cellular Movement |
| **B** | **ADTRP**, APP, **B4GALNT3**, CACHD1, **CDHR2**, ECI2, ETHE1, **ETV7**, **FAM90A1**, **GBP5**, GNPTAB, HPCA, HTR2C, IL10RA, **KIAA1598**, KLHL41, **MAOA**, MAOB, MYO7B, MYPN, **NEB**, PPARA, **PRSS21**, **PRUNE2**, Ppap2a, **RPL39L**, RPL7L1, **SCAMP5**, **SDC3**, SRC, STYXL1, TMOD4, UBC, **VWA8**, XIRP2 | 30 | 15 | Cell-To-Cell Signaling and Interaction, Drug Metabolism, Energy Production |
| **C** | **ADAMTS1**, ADCY, **AKR1C1/AKR1C2**, AMPK, Akt, **CYP4F2**, Cg, **EGR2**, ERK, **FADS2**, **FBXW8**, **FLT3**, FSH, Focal adhesion kinase, **G0S2**, **GNLY**, Hdac, Hsp70, Hsp90, IL1, Insulin, **KCNMA1**, Lh, **NRG1**, PDGF BB, **PID1**, **PTGDS**, Pka, Pkc(s), Ras, **SCT**, **SLC22A17**, Vegf, caspase, p85 (pik3r) | 30 | 15 | Lipid Metabolism, Small Molecule Biochemistry, Behavior |
| **D** | **AK5**, **AKR1C1/AKR1C2**, ARFGAP3, ASS1, **BATF2**, C1R, **C4BPA**, CALB2, CAPG, **CARD17**, **CCL23**, **CD177**, CD276, CEBPA, CSF3, CTNNB1, CTNND2, Cebp, Collagen type VI, **DACT1**, DACT2, DACT3, DSPP, **GPC4**, HLA-F, **HRK**, IFNG, KLK8, LILRA2, OSM, **PDCD1LG2**, PRTN3, SLC16A1, **VLDLR**, WNT5A | 23 | 12 | Organismal Injury and Abnormalities, Tissue Morphology, Cellular Growth and Proliferation |

*A table of the names of transcripts included in the top 4 networks derived from IPA, as well as the top diseases and functions of these transcripts. Neutrophil transcripts in bold were differentially expressed between patients with and without intracranial aneurysm (p-value<0.05). Each network’s p-score was derived from its p-value [p-score = -Log10 (p-value)] calculated by the Fisher’s exact test. For a network with a p-score of 10, the odds of generating this network by chance alone is less than 1 out of 10^10^.
