# Supplementary material for: Circulating neutrophil transcriptome may reveal intracranial aneurysm signature
Source: PLoS One. 2018 Jan 17;13(1):e0191407. doi: 10.1371/journal.pone.0191407 (PMC5771622; doi:10.1371/journal.pone.0191407)
Supplement: S6 Table — *(IA = intracranial aneurysm, SE = standard error, Q = quartile). (DOCX) [file pone.0191407.s008.docx]

**S6 Table. Clinical characteristics of the unpaired cohort of 5 patients with intracranial aneurysms and 5 control subjects without intracranial aneurysms (confirmed on imaging)***

|  | **Patients with IA**  **(n=5)** | **Patients without**  **IA (n=5)** |
| --- | --- | --- |
| **Age (years) (Mean ± SE)** | 56.8±3.95 | 48.8±6.65 |
| **Age (years) [Median (Q1/Q3)]** | 56 (53/57) | 54 (51/55) |
| **Sex** |  |  |
| *Female* | 60% | 20% |
| **Current smoker** |  |  |
| *Yes* | 40% | 0% |
| **Comorbidities** |  |  |
| *Hypertension* | 40% | 40% |
| *Hyperlipidemia* | 20% | 20% |
| *Heart disease* | 20% | 0% |
| *Previous stroke* | 0% | 0% |
| *Diabetes mellitus* | 20% | 20% |
| *Osteoarthritis* | 20% | 20% |

*(IA=intracranial aneurysm, SE=standard error, Q=quartile)
