# Supplementary material for: Circulating neutrophil transcriptome may reveal intracranial aneurysm signature
Source: PLoS One. 2018 Jan 17;13(1):e0191407. doi: 10.1371/journal.pone.0191407 (PMC5771622; doi:10.1371/journal.pone.0191407)
Supplement: S7 Table — *Aneurysm size ranged from 3.5 mm to 7 mm. Five of 6 IAs (83%) were classified as small (greatest diameter <7mm) and 1 (17%) was classified as large (greatest diameter ≥7 mm). The aneurysms were situated at various locations in the Circle of Willis, with most being in the anterior vasculature (ACA and MCA). (ACA = anterior cerebral artery, AComA = anterior communicating artery, BT = basilar terminus, CT = computed tomography, DSA = digital subtraction angiography, IA, intracranial aneurysm, MCA = middle cerebral artery, MRA = magnetic resonance angiography, MRI = magnetic resonance imaging). (DOCX) [file pone.0191407.s009.docx]

**S7 Table**. **Characteristics of 6 intracranial aneurysms in the replication group of 5 patients with IAs (one patients had multiple intracranial aneurysms)***

| **IA Patient no.** | **IA**  **Size**  **(mm)** | **IA**  **Location** | **Presence of**  **Additional IAs** | **Family**  **History of IA** | **Indications for DSA** |
| --- | --- | --- | --- | --- | --- |
| 12 | 2 | ACA | No | No | Follow-up imaging of known IA |
| 13 | 1.4 | MCA | No | No | Incidental finding on MRI indicated possible IA |
| 14 | 3.7 | AComA | No | No | Incidental finding on CT for headache indicated possible IA |
| 15 | 7 | MCA | Yes: +1 (3.5 mm ACA) | No | Follow-up imaging of known IA |
| 16 | 3.9 | BT | no | No | Incidental finding on MRA for headache indicated possible IA |

*Aneurysm size ranged from 3.5 mm to 7 mm. Five of 6 IAs (83%) were classified as small (greatest diameter <7mm) and 1 (17%) was classified as large (greatest diameter ≥7 mm). The aneurysms were situated at various locations in the Circle of Willis, with most being in the anterior vasculature (ACA and MCA). (ACA=anterior cerebral artery, AComA=anterior communicating artery, BT=basilar terminus, CT=computed tomography, DSA=digital subtraction angiography, IA, intracranial aneurysm, MCA=middle cerebral artery, MRA=magnetic resonance angiography, MRI=magnetic resonance imaging)
