# Supplementary material for: Circulating neutrophil transcriptome may reveal intracranial aneurysm signature
Source: PLoS One. 2018 Jan 17;13(1):e0191407. doi: 10.1371/journal.pone.0191407 (PMC5771622; doi:10.1371/journal.pone.0191407)
Supplement: S8 Table — *(M = male, F = female, Y = yes, N = no, HT = hypertension, HL = hyperlipidemia, CAD = coronary artery disease, S Hx = stroke history, DM = diabetes mellitus, OA = osteoarthritis). (DOCX) [file pone.0191407.s010.docx]

**S8 Table. Deidentified patient metadata.***

|  | **ID** | **Class** | **Age** | **Sex** | **Smoker** | **HT** | **HL** | **CAD** | **S Hx** | **DM** | **OA** |
| --- | --- | --- | --- | --- | --- | --- | --- | --- | --- | --- | --- |
|  |  |  |  | (M/F) | (Y/N) | Presence (1=yes, 0=no) | | | | | |
| ***Original Cohort*** | | |  |  |  |  |  |  |  |  |  |
|  | C1 | Control | 71 | M | Y | 1 | 1 | 1 | 0 | 0 | 0 |
|  | C2 | Control | 83 | F | N | 1 | 1 | 0 | 0 | 0 | 1 |
|  | C3 | Control | 62 | M | N | 1 | 1 | 1 | 0 | 0 | 1 |
|  | C4 | Control | 77 | M | N | 1 | 1 | 0 | 0 | 0 | 0 |
|  | C5 | Control | 36 | F | N | 0 | 0 | 0 | 0 | 0 | 0 |
|  | C6 | Control | 71 | F | N | 1 | 0 | 0 | 0 | 0 | 0 |
|  | C7 | Control | 44 | M | N | 1 | 0 | 0 | 1 | 1 | 0 |
|  | C8 | Control | 58 | F | N | 1 | 1 | 0 | 0 | 1 | 0 |
|  | C9 | Control | 72 | F | N | 1 | 0 | 0 | 0 | 1 | 0 |
|  | C10 | Control | 70 | M | N | 1 | 0 | 1 | 0 | 1 | 1 |
|  | C11 | Control | 68 | F | Y | 0 | 1 | 0 | 0 | 0 | 0 |
|  | A1 | Aneurysm | 54 | M | N | 1 | 0 | 0 | 0 | 0 | 0 |
|  | A2 | Aneurysm | 61 | F | Y | 0 | 1 | 0 | 0 | 0 | 0 |
|  | A3 | Aneurysm | 75 | M | N | 1 | 0 | 0 | 0 | 0 | 0 |
|  | A4 | Aneurysm | 66 | F | N | 0 | 1 | 0 | 0 | 1 | 1 |
|  | A5 | Aneurysm | 67 | M | N | 1 | 0 | 1 | 0 | 0 | 0 |
|  | A6 | Aneurysm | 80 | M | N | 0 | 0 | 1 | 0 | 0 | 0 |
|  | A7 | Aneurysm | 68 | F | N | 1 | 1 | 0 | 0 | 0 | 1 |
|  | A8 | Aneurysm | 82 | F | N | 1 | 1 | 0 | 0 | 1 | 0 |
|  | A9 | Aneurysm | 54 | F | Y | 0 | 0 | 0 | 0 | 0 | 0 |
|  | A10 | Aneurysm | 69 | F | N | 1 | 0 | 0 | 0 | 0 | 1 |
|  | A11 | Aneurysm | 60 | F | N | 1 | 1 | 0 | 0 | 0 | 0 |
| ***Replication Cohort*** | | | |  |  |  |  |  |  |  |  |
|  | C12 | Control | 61 | M | N | 1 | 0 | 0 | 0 | 0 | 1 |
|  | C13 | Control | 54 | M | N | 1 | 1 | 0 | 0 | 1 | 0 |
|  | C14 | Control | 51 | M | N | 0 | 0 | 0 | 0 | 0 | 0 |
|  | C15 | Control | 23 | F | N | 0 | 0 | 0 | 0 | 0 | 0 |
|  | C16 | Control | 55 | M | N | 0 | 0 | 0 | 0 | 0 | 0 |
|  | A12 | Aneurysm | 53 | F | Y | 0 | 0 | 0 | 0 | 0 | 0 |
|  | A13 | Aneurysm | 56 | M | N | 1 | 0 | 0 | 0 | 0 | 0 |
|  | A14 | Aneurysm | 71 | F | N | 1 | 1 | 1 | 0 | 1 | 1 |
|  | A15 | Aneurysm | 47 | M | Y | 0 | 0 | 0 | 0 | 0 | 0 |
|  | A16 | Aneurysm | 57 | F | N | 0 | 0 | 0 | 0 | 0 | 0 |

*(M=male, F=female, Y=yes, N=no, HT=hypertension, HL=hyperlipidemia, CAD=coronary artery disease, S Hx=stroke history, DM=diabetes mellitus, OA=osteoarthritis)
